# Reconstruction Modeling and Validation of Brown Croaker (*Miichthys miiuy*) Vocalizations Using Wavelet-Based Inversion and Deep Learning

**DOI:** 10.3390/s25196178

**Published:** 2025-10-06

**Authors:** Sunhyo Kim, Jongwook Choi, Bum-Kyu Kim, Hansoo Kim, Donhyug Kang, Jee Woong Choi, Young Geul Yoon, Sungho Cho

**Affiliations:** 1 Sea Power Reinforcement Security Research Department, Korea Institute of Ocean Science & Technology (KIOST), Busan 49111, Republic of Korea; sunhyo@kiost.ac.kr (S.K.); kimbk91@kiost.ac.kr (B.-K.K.); hskim@kiost.ac.kr (H.K.); dhkang@kiost.ac.kr (D.K.); ygyoon@kiost.ac.kr (Y.G.Y.); 2 Department of Marine Science and Convergence Engineering, Hanyang University ERICA, Ansan 15588, Republic of Korea; jong238@hanyang.ac.kr; 3 School of Defense Intelligence and Information Convergence Engineering, Hanyang University ERICA, Ansan 15588, Republic of Korea; choijw@hanyang.ac.kr

**Keywords:** brown croaker vocalization, passive acoustic monitoring (PAM), bioacoustic signal reconstruction, waveform similarity

## Abstract

**Highlights:**

- We developed a wavelet-based framework using fk14 synthesis and particle swarm optimization (PSO) to reconstruct brown croaker vocalizations.
- Parameter sensitivity analysis identified delay and scale as key factors, enabling >98% waveform similarity in marine bioacoustic reconstruction.
- A ResNet-18-based Siamese network validated near-unity similarity (~0.9996) and evaluated across varying SNR conditions.
- This method enables high-fidelity bioacoustic signal synthesis for passive monitoring and machine learning data augmentation.

**Abstract:**

Fish species’ biological vocalizations serve as essential acoustic signatures for passive acoustic monitoring (PAM) and ecological assessments. However, limited availability of high-quality acoustic recordings, particularly for region-specific species like the brown croaker (*Miichthys miiuy*), hampers data-driven bioacoustic methodology development. In this study, we present a framework for reconstructing brown croaker vocalizations by integrating fk14 wavelet synthesis, PSO-based parameter optimization (with an objective combining correlation and normalized MSE), and deep learning-based validation. Sensitivity analysis using a normalized Bartlett processor identified delay and scale (length) as the most critical parameters, defining valid ranges that maintained waveform similarity above 98%. The reconstructed signals matched measured calls in both time and frequency domains, replicating single-pulse morphology, inter-pulse interval (IPI) distributions, and energy spectral density. Validation with a ResNet-18-based Siamese network produced near-unity cosine similarity (~0.9996) between measured and reconstructed signals. Statistical analyses (95% confidence intervals; residual errors) confirmed faithful preservation of SPL values and minor, biologically plausible IPI variations. Under noisy conditions, similarity decreased as SNR dropped, indicating that environmental noise affects reconstruction fidelity. These results demonstrate that the proposed framework can reliably generate acoustically realistic and morphologically consistent fish vocalizations, even under data-limited scenarios. The methodology holds promise for dataset augmentation, PAM applications, and species-specific call simulation. Future work will extend this framework by using reconstructed signals to train generative models (e.g., GANs, WaveNet), enabling scalable synthesis and supporting real-time adaptive modeling in field monitoring.

## 1. Introduction

Biological sounds produced by fish are crucial for underwater ecological monitoring and species classification. Many teleost fishes emit distinct acoustic signals for activities such as mating, territorial defense, and group coordination. These vocalizations often feature species-specific temporal and spectral characteristics, making them valuable indicators for passive acoustic monitoring (PAM) in marine environments [1,2,3,4].

Among sound-producing fish, the brown croaker (*Miichthys miiuy*) is notable for generating low-frequency pulsed calls through contractions of specialized sonic muscles and the swim bladder. These calls are mainly observed during the spawning season and are believed to aid in mate attraction and synchronize reproductive behavior. Owing to their acoustic prominence and ecological importance, brown croaker vocalizations are of considerable interest for both marine biological research and underwater acoustic system development [5,6].

However, access to high-quality observational data for the brown croaker remains limited, especially in the southwestern coastal waters of the Korean Peninsula, where the species is endemic. This lack of empirical recordings substantially hinders the development of data-driven approaches for acoustic signal analysis, species classification, bioacoustic modeling, and AI-based underwater acoustic technologies. Although numerous studies have explored sinusoidal and wavelet-based techniques for synthesizing marine biological sounds [7,8,9,10,11,12], only a few have employed advanced optimization methodologies to achieve high-fidelity waveform reconstruction. Moreover, modeling approaches that preserve species-specific acoustic characteristics and assess similarity are limited.

Building upon previous research, we combined wavelet-based signal synthesis with particle swarm optimization (PSO) to accurately reconstruct brown croaker vocalizations. We conducted a sensitivity analysis of wavelet parameters to model diverse vocalization patterns while maintaining the species’ distinctive features. The synthesized waveforms were evaluated against measured calls through time- and frequency-domain analyses. Additionally, a deep convolutional neural network (ResNet-18), trained on recorded brown croaker calls, was utilized to quantitatively measure the similarity between the reconstructed signals and measured calls.

The remainder of this manuscript is organized as follows: Section 2 outlines the acoustic characteristics of brown croaker vocalizations, including data acquisition methods and feature extraction procedures. Section 3 details the wavelet-based modeling framework, including parameter optimization using PSO and sensitivity analysis for modeling signal variability. Section 4 presents the experimental results, encompassing waveform reconstruction performance and similarity validation using a ResNet-18 classifier-based deep learning approach. Finally, Section 5 summarizes the findings, discusses the implications of the results, and explores potential applications in marine bioacoustics.

## 2. Acoustic Characteristics of the Brown Croaker Vocalizations

### 2.1. Acquisition and Analysis Methods of Brown Croaker Vocalizations

Acoustic signals from 150 adult brown croakers, each measuring between 60 and 80 cm in total length, were recorded as the fish freely swam within a sea cage located in the South Sea of Korea. The Maritime Test and Evaluation Station at the Korea Institute of Ocean Science and Technology (KIOST) conducted these recordings.

An underwater self-recording hydrophone (SM3M; Wildlife Acoustics, Inc., Maynard, MA, USA) was deployed to continuously capture acoustic signals over a 13 d period, from 18 to 30 September 2023. The hydrophone was set with a receiving voltage sensitivity of −164.6 dB re 1 V/µPa, 0 dB gain, and a sampling frequency of 48 kHz. Recorded data were stored as 10 min digital audio segments (*.wav). Subsequent analysis of brown croaker vocalizations was performed using a custom-developed acoustic analysis program in MATLAB 2025a (MathWorks, USA), enabling detailed signal processing and feature extraction. Signals with amplitudes below the background noise level and those containing overlapping calls were excluded from the analysis. Brown croaker vocalizations comprised single-pulse calls and multipulse train calls, the latter consisting of a series of consecutive pulse events emitted in a temporally organized manner with relatively consistent inter-pulse intervals (IPIs).

### 2.2. Acoustic Feature Analysis of Brown Croaker Vocalizations

Specifically, the acoustic characteristics—the amplitude of single pulses and the IPI of pulse trains—were extracted and utilized as key parameters for modeling the reconstruction of biological sounds. To characterize the acoustic features of the brown croaker, 522 single-pulse calls with clear, unclipped waveforms were extracted. These calls were time-aligned to illustrate the temporal characteristics of the vocalizations.

**Figure 1:**
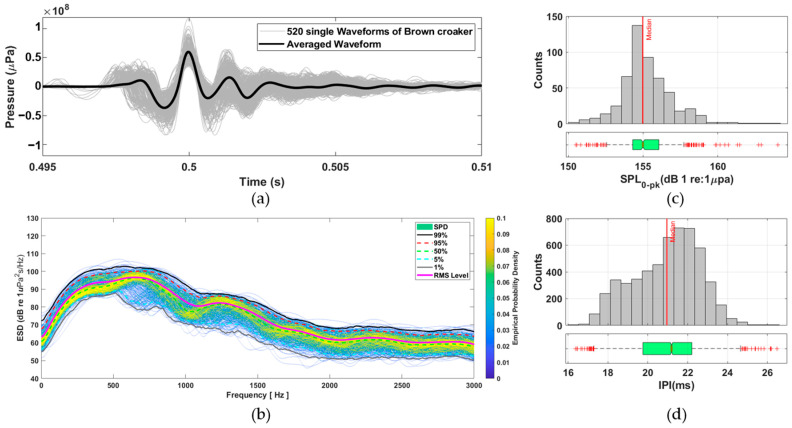
Acoustic feature extraction from brown croaker calls. (**a**) Time-domain superposition of 522 single-pulse calls (gray) with the averaged call overlaid (bold black). (**b**) Energy spectral density analysis with percentile curves and a probability density color map. (**c**) Distribution of zero-to-peak sound pressure level (SPL_0–pk_) with a median line and boxplot. (**d**) Distribution of inter-pulse intervals extracted from 1157 pulse train calls, with median and boxplot visualizations.

As shown in Figure 1a, the waveforms (gray lines) exhibit highly consistent structures across samples, marked by a rapid onset, a prominent positive pressure peak, and subsequent damped oscillations. The bold black line represents the averaged waveform, exemplifying the typical pulse morphology of brown croaker vocalizations.

In the frequency domain, energy spectral density (ESD) analysis was performed on the same 522 single pulses (Figure 1b). The color map illustrates the empirical probability density of spectral energy in each frequency bin, with warmer colors indicating higher probability. Superimposed percentile curves (1%, 5%, 50%, 95%, 99%) highlight the spectral variability across the dataset. Most of the acoustic energy was concentrated between 300 and 900 Hz, with a distinct spectral peak around 500–600 Hz. Quantitative distributions of the extracted acoustic parameters are presented in Figure 1c,d. Figure 1c shows the distribution of zero-to-peak sound pressure levels (SPL_0–pk_) from 522 individual single-pulse calls, yielding a median of 154.99 dB, with the 25th and 75th percentiles at 154.30 dB and 156.06 dB, respectively. Figure 1d displays the distribution of IPI, derived from 1157 pulse train calls, with a median of 20.95 ms and 25th and 75th percentiles of 19.75 ms and 22.21 ms, respectively. The pulse amplitude and IPI were identified as key variables for subsequent call reconstruction modeling.

## 3. Signal Modeling Framework for Brown Croaker Vocalization Reconstruction

To accurately reconstruct the vocalizations of the brown croaker (*Miichthys miiuy*), a signal modeling framework was developed, integrating wavelet-based synthesis, parameter optimization, and sensitivity analysis. Specifically, an acoustic reconstruction algorithm employed fk14 wavelets chosen for their morphological resemblance to the natural pulse shapes observed in empirical recordings. The synthesized waveform was created by summing multiple wavelet components, each characterized by three parameters: amplitude, delay, and scale (length) [8]. PSO was utilized to identify optimal parameter values, minimizing the discrepancy between the measured and synthesized waveforms. To improve reliability and robustness, the optimization objective function was defined as a composite of the correlation coefficient and the normalized mean squared error (MSE). Throughout the iterative PSO process, each particle adjusted its parameters based on personal and global best solutions, progressively improving synthesis accuracy [13,14,15,16].

Furthermore, sensitivity analysis was performed to evaluate the impact of individual wavelet parameters on the reconstructed signal, thereby facilitating the generation of diverse yet biologically consistent waveforms. This comprehensive methodology enables robust modeling of brown croaker vocalizations in data-scarce environments and underpins data-driven bioacoustic analysis.

### 3.1. Wavelet-Based Reconstruction of Brown Croaker Vocalizations

The single-pulse call of the brown croaker is characterized by low-amplitude positive and negative pre-peaks, followed by a prominent high-amplitude positive peak and a subsequent single-peak decay. To effectively capture the morphological structure, the fk14 wavelet from the Fejér–Korovkin family, featuring a filter length of 14, was selected for signal modeling [13]. This wavelet exhibits compact support, symmetry, smooth decay, and strong localization in both time and frequency domains, making it ideal for representing the transient and oscillatory nature of fish bioacoustic signals.

Using this framework, the brown croaker call was reconstructed by generating individual wavelet components through parameterizing amplitude, delay, and scale length applied to the predefined mother wavelet (fk14). These components were then summed to synthesize the complete waveform. The final synthesized waveform S(t) is represented as a linear combination of N wavelets, with each component individually fine-tuned to replicate the temporal and spectral features observed in the recorded brown croaker calls.

(1) $$S(t)=\sum_{i=1}^{N}A_{i}\psi \left(\frac{t-\tau_{i}}{\sigma_{i}}\right),$$

where $A_{i}$, $\tau_{i}$, and $\sigma_{i}$ represent the amplitude, delay, and scale (length) parameters of the i wavelet component, respectively, and ψ denotes the fk14 wavelet function used as the mother wavelet.

### 3.2. Parameter Optimization Using Particle Swarm Optimization (PSO)

The brown croaker call was reconstructed using PSO, which identified the optimal wavelet parameters to replicate the temporal characteristics of the recorded calls. PSO, a stochastic search algorithm inspired by social behaviors of animals like bird flocking and fish schooling, seeks optimal solutions efficiently. Widely adopted for its rapid convergence, robustness, and effectiveness in dynamic optimization problems [14,15,16,17,18,19], PSO was employed to determine the wavelet parameters that minimize the error between the synthesized waveform and the empirically observed brown croaker call.

In PSO, particles represent individual wavelet parameters (wavelet length, wavelet amplitude, wavelet delay) and collectively form the swarm. The algorithm begins by generating a swarm of candidate solutions, each corresponding to a unique combination of wavelet parameters. During optimization, particles iteratively explore the parameter space, updating their positions and velocities based on their personal best experiences and the global best solution of the swarm.

(2) $$v_{i,\;d}\left(n+1\right)=v_{i,\;d}\left(n\right)+c_{1}r_{1}\left[p_{i,\;d}\left(n\right)-x_{i,\;d}\left(n\right)\right]+c_{2}r_{2}\left[p_{g,\;d}\left(n\right)-x_{i,\;d}\left(n\right)\right],$$

(3) $$x_{i,\;d}\left(n+1\right)=x_{i,\;d}\left(n\right)+v_{i,\;d}\left(n+1\right).$$

In the PSO algorithm, $x_{i,\;d}\left(n\right)$ and $v_{i,\;d}\left(n\right)$ denote the position (wavelet parameter) and velocity of the i-th particle in the d-th dimension at iteration n, respectively. $p_{i,\;d}\left(n\right)$ denotes the personal best position of the particle up to iteration n, while $p_{g,\;d}\left(n\right)$ represents the global best position identified by the swarm.

**Figure 2:**
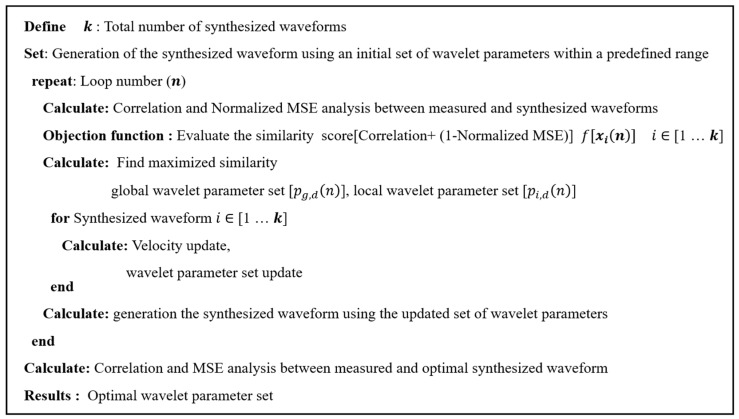
Pseudocode of the PSO algorithm for the search of the optimal wavelet parameter set for the synthesized waveform. The objective function was defined as a composite of correlation and normalized MSE to improve reliability and robustness.

The constants $c_{1}$ and $c_{2}$ are acceleration coefficients; and $r_{1}$, $r_{2}$ are uniformly distributed random numbers between 0 and 1. To improve the reliability and robustness of the optimization process, the objective function was defined as a composite of correlation and normalized MSE, rather than correlation alone. Figure 2 presents the pseudocode of the PSO algorithm used to optimize the wavelet parameter set.

The PSO was set with a population size of 1000 and a maximum of 80 iterations. The search space was defined as follows: wavelet amplitude [0.0, 1.0], wavelet length [10, 1000], and wavelet delay [−100, 100].

**Figure 3:**
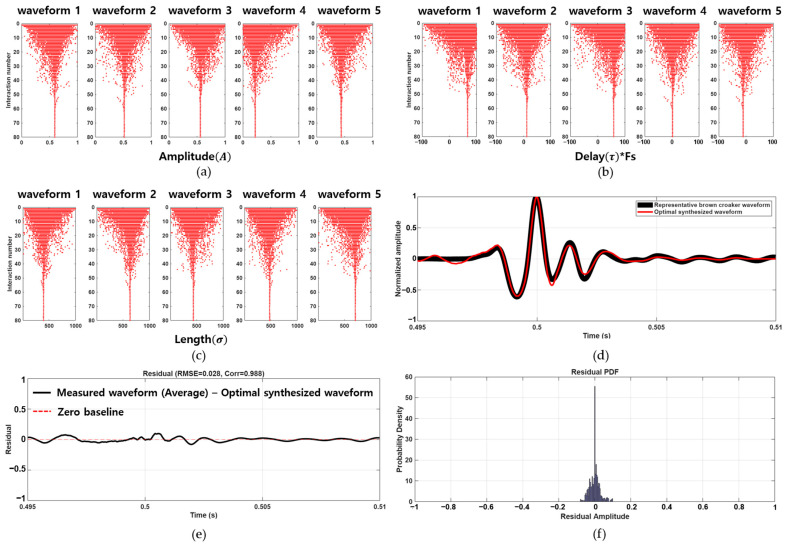
Convergence trajectories of (**a**) amplitude (*A*), (**b**) delay (*τ* × Fs), and (**c**) length (*σ*) parameters for each of the five wavelet components across 80 iterations of the PSO algorithm. (**d**) Time-domain comparison between the representative measured brown croaker call (black) and the optimally synthesized waveform (magenta). (**e**) Residual waveform with RMSE = 0.028 and correlation = 0.988. (**f**) Residual PDF showing small errors.

Empirical results showed that the correlation and the normalized MSE between the measured and synthesized waveforms plateaued after 80 iterations, indicating the convergence of the optimization process. As shown in Figure 3a–c, the optimization trajectories of the wavelet parameters amplitude (*A*), delay (*τ* × Fs), and length (*σ*) for five synthesized waveforms. The gradual clustering of parameter values demonstrates the algorithm’s capacity to converge toward an optimal solution with increasing iterations. Figure 3d compares the time-domain waveforms of the representative measured brown croaker call (black line) and the corresponding synthesized waveform (red line) reconstructed using the optimized parameters, showing a close agreement in the time domain. The residual analysis (Figure 3e,f) confirmed that the differences between measured and synthesized signals were minimal, yielding an RMSE of 0.028 and a correlation of 0.988.

Table 1 summarizes the optimal parameter sets for the five wavelet components employed in reconstructing the representative brown croaker waveform. The parameters include amplitude (normalized), delay (in sample points, equivalent to milliseconds, representing the waveform’s central position), and length (in samples, representing the temporal spread of the waveform). The synthesized waveform successfully captures the key temporal features of the measured call, such as the initial low-amplitude peaks, the dominant positive peak, and the subsequent damped oscillations, thereby validating the effectiveness of the proposed modeling approach.

### 3.3. Sensitivity Analysis for Defining Parameter Ranges and Modeling Signal Variability

To generate diverse yet biologically realistic brown croaker vocalizations, a signal modeling approach was employed to simulate the natural variability observed in recorded acoustic data. This approach was particularly important for constructing training datasets for AI-based classification models and for assessing the robustness of underwater acoustic systems under varying signal conditions. To accomplish this, a sensitivity analysis was conducted on the inverted wavelet parameters derived from the optimal synthesis process [19,20]. The objective was to identify the permissible perturbation range for each parameter amplitude, delay, and length that preserves the core temporal and spectral characteristics of the measured calls, thereby enabling controlled diversification of the synthesized waveforms without compromising their biological plausibility.

To evaluate the sensitivity of the wavelet parameters used in reconstructing the brown croaker waveform, a parameter-wise sensitivity analysis was conducted using a normalized Bartlett processor ∅, as defined in [21,22]:

(4) $$\emptyset \;=\;1-\frac{1}{L}\sum_{i=1}^{L}\frac{R_{N}^{T}C_{N}R_{N}\;}{R_{N}^{T}R_{N}}.$$

Here, $R_{N}$ represents the normalized Fourier-transformed synthesized waveform corresponding to a specific wavelet parameter configuration, and $C_{N}$ denotes the normalized cross-spectral density matrix derived from the Fourier-transformed measured brown croaker waveform. T denotes the transpose. The sensitivity of each wavelet parameter amplitude (*A*), delay (*τ* × Fs), and length (*σ*) was assessed by systematically varying the target parameter within its predefined search range while keeping the other parameters at their optimal values. This approach isolates the contribution of each parameter to the accuracy of waveform inversion.

**Figure 4:**
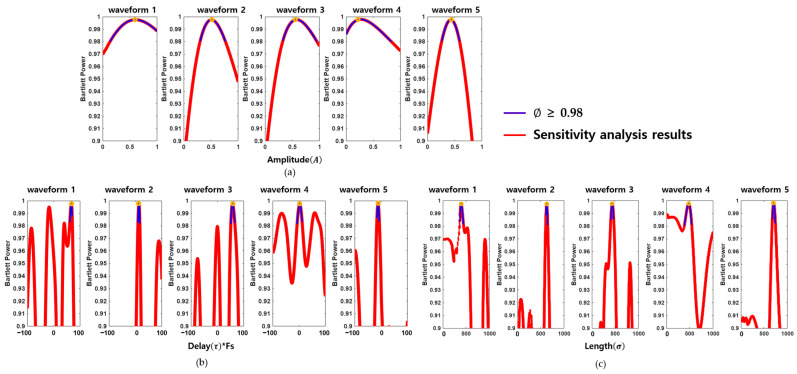
Sensitivity analysis of wavelet parameters used in signal modeling. Plots in (**a**–**c**) show Bartlett power values for sensitivity plots for each wavelet parameter, where Bartlett power remains above 0.98, indicating robust synthesis similarity for each waveform.

As displayed in Figure 4, the results demonstrate that the objective function ∅ is particularly sensitive to changes in delay and length parameters (Figure 4b,c), whereas amplitude (Figure 4a) has a relatively minor impact. This highlights the importance of precisely tuning the delay and length parameters during signal modeling while allowing greater flexibility in amplitude variation.

Additionally, Table 2 outlines the effective modeling ranges, defined as intervals where ∅ ≥ 0.98 for each parameter across five representative waveforms. These parameter intervals provide practical limits for generating signal variants that maintain both biological realism and high similarity to the measured brown croaker calls. Such modeling flexibility is crucial for producing diverse yet accurate synthetic waveforms for subsequent applications in acoustic simulation, classification training.

### 3.4. Sensitivity-Based Signal Modeling Results

To evaluate the effectiveness of our proposed signal modeling approach, we compared synthesized brown croaker waveforms with measured calls in both time and frequency domains. The reconstructed signals were generated using wavelet-based inversion coupled with sensitivity-constrained parameter ranges derived from prior analyses.

**Figure 5:**
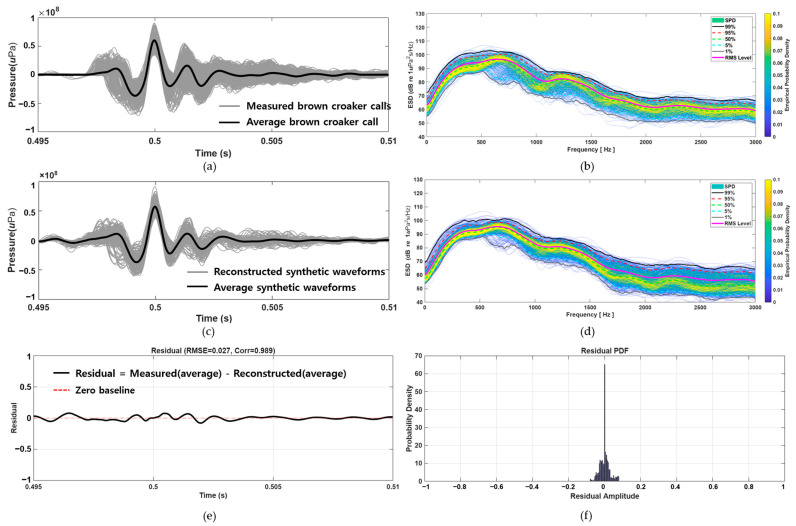
Time and frequency domain comparison between measured calls and reconstructed signals. (**a**) Superimposed measured brown croaker calls (gray) and their average (black). (**b**) ESD of measured calls with percentile curves and RMS level. (**c**) Superimposed reconstructed synthetic waveforms (gray) and their average (black). (**d**) ESD of synthetic waveforms showing close alignment with measured calls. (**e**) Residual waveform with RMSE = 0.027 and correlation = 0.989. (**f**) Probability density function (PDF) of residual amplitudes, confirming small reconstruction errors.

In the time domain, the superimposed waveforms and their averages indicate that the reconstructed signals successfully reproduce the key morphological features of the measured brown croaker calls. Specifically, the models accurately recreated the low-amplitude pre-peaks, prominent central peak, and post-peak damped oscillations (Figure 5a,c). These findings suggest that the synthesis method maintains the structural integrity of the biologically observed pulses. The residual analysis (Figure 5e,f) further confirmed that the differences between measured and reconstructed signals were minimal, yielding an RMSE of 0.027 and a correlation of 0.989. These results demonstrate that the proposed synthesis method preserves the structural integrity of the biologically observed pulses. In the frequency domain, the ESD of the reconstructed signals closely aligned with that of the measured calls. As shown in Figure 5b,d, most of the acoustic energy was concentrated between 300 and 900 Hz, with both datasets displaying a distinct spectral peak around 500–700 Hz. Additionally, the percentile curves and root mean square (RMS) levels were nearly identical, further validating the spectral robustness of the reconstructed signals. Collectively, these results confirm the efficacy of the proposed signal modeling framework in generating acoustically realistic and morphologically consistent brown croaker vocalizations.

**Figure 6:**
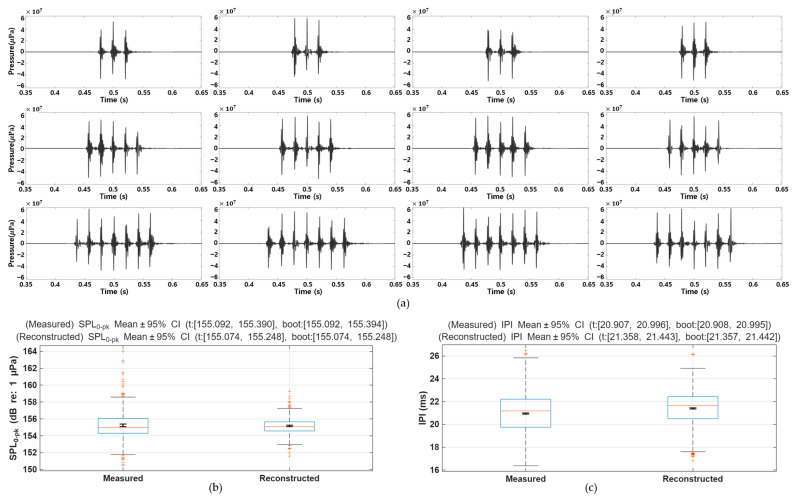
Reconstructed pulse train waveforms that replicate key acoustic features, using the proposed modeling framework. Reconstructed pulse train waveforms replicating key acoustic features of brown croaker vocalizations using the proposed modeling framework. (**a**) Examples of reconstructed pulse trains generated by concatenating individually synthesized pulses based on empirically derived inter-pulse intervals (IPI). (**b**) Boxplots of measured and reconstructed zero-to-peak sound pressure level (SPL_0–pk_) with mean ± 95% confidence intervals (CI), showing no statistically significant difference. (**c**) Boxplots of measured and reconstructed IPI with mean ± 95% CI, indicating a slight but consistent shift.

Figure 6a presents the modeling results of pulse-train calls constructed to replicate the temporal and amplitude characteristics of natural brown croaker vocalizations. The synthetic waveforms were generated by concatenating individually reconstructed single pulses according to the empirically IPI. Statistical analysis confirmed that the measured IPI had a mean of 20.95 ms with a 95% confidence interval (CI) of [20.91, 21.00] ms, while the reconstructed IPI had a mean of 21.40 ms with a 95% CI of [21.36, 21.44] ms. Similarly, the zero-to-peak sound pressure level (SPL_0–pk_) of the measured pulses averaged 155.24 dB re 1 µPa (95% CI: [155.09, 155.39] dB), compared with 155.16 dB re 1 µPa (95% CI: [155.07, 155.25] dB) for the reconstructed pulses. The corresponding boxplots (Figure 5c and Figure 6a) show that the distributions of SPL_0–pk_ and IPI values between measured and reconstructed signals exhibit close agreement, with no statistically significant difference in SPL_0–pk_ and only a slight shift in IPI. Collectively, these results demonstrate that the proposed synthesis framework preserves both the rhythmic timing and amplitude variability of brown croaker pulse trains, validating its effectiveness in generating biologically realistic acoustic sequences.

To evaluate computational feasibility, we measured the runtime of the proposed framework. Reconstruction of 500 signals required approximately 17 s on a standard desktop workstation (Intel i7 CPU, 32 GB RAM), corresponding to an average processing time of ~34 ms per signal. These results demonstrate that the algorithm operates efficiently and can be applied to large-scale data augmentation tasks, with potential for further acceleration using GPU or parallel computing.

## 4. Deep Learning-Based Similarity Evaluation

A Siamese network based on a ResNet-18 (Residual Network-18) backbone, a convolutional neural network architecture, was employed to evaluate the similarity and validity of the reconstructed brown croaker waveforms. The Siamese network consists of two identical weight-sharing subnetworks and ultimately outputs the cosine similarity computed between the 512-dimensional embeddings produced by ResNet-18 [23]. The model contains 11,446,336 parameters, and the latent representations extracted at the end of the classifier are high-dimensional embeddings that compress and encapsulate the fundamental characteristics of each signal. Cosine similarity was then used to assess the directional alignment of these vectors, with values approaching 1 indicating greater similarity between the measured calls and the reconstructed signals.

**Figure 7:**
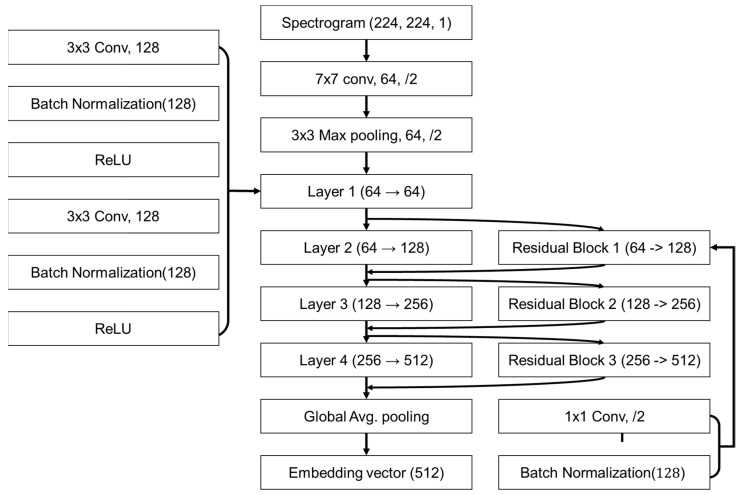
Modified ResNet-18 architecture used for similarity analysis of reconstructed signals.

Figure 7 depicts the modified ResNet-18 architecture. The input spectrogram (224 × 224 × 1) is first processed using a 7 × 7 convolutional layer followed by a 3 × 3 max pooling layer, and then passed through four sequential residual layers. Each residual block increases the channel dimension while preserving learned features via skip connections. The final feature representation is obtained via global average pooling. In addition, an auxiliary convolutional module (left) and a 1 × 1 convolution block (right) are incorporated for feature enhancement and dimensionality adjustment, respectively. The dataset consisted of 1023 measured samples distributed across chains: chain 1 (n = 519), chain 3 (n = 158), chain 5 (n = 118), chain 7 (n = 79), chain 9 (n = 92), chain 11 (n = 53), and chain 13 (n = 4). The training and validation datasets were obtained by splitting the measured calls into a 7:3 ratio with no overlap. For similarity evaluation, a separate test set consisted of reconstructed signals and low-SNR versions of the measured calls at SNRs of 15 dB and 10 dB. All inputs were min–max-normalized to [0, 1], mapping 60 dB to 0 and 120 dB to 1. For the reconstructed signals, 500 samples were generated per chain. The 15 dB and 10 dB SNR sets were derived from the measured calls, so the numbers of calls and the per-chain counts are identical to those of the measured calls.

For training and testing, input pairs were generated on a per-chain basis from the dataset by forming positive and negative pairs. Labels were assigned as 0 for positive pairs, comprising two signals from the same class, and 1 for negative pairs, consisting of signals from different classes. A positive pair was created by independently sampling two examples from a single class, while a negative pair was constructed by selecting two different classes and sampling one example from each.

Optimization was performed using the Adam (Adaptive Moment Estimation) algorithm [24] with a learning rate of 0.00001 to ensure stable training. The loss function for similarity evaluation was the contrastive loss [25] with a margin of 0.3. Each iteration employed a mini-batch size of 64, and evaluations were conducted every 100 steps, with early stopping applied if the loss did not improve for five consecutive evaluations. To assess the preservation of intrinsic characteristics, the mean cosine similarity was calculated between the latent vectors of measured calls, reconstructed signals, and low-SNR signals within the same class. This analysis facilitated both quantitative and qualitative assessments of the reconstructed signals by examining the class separability and representational similarity to the measured calls, thereby validating the reliability of the proposed reconstruction framework.

Table 3 presents the cosine similarity and performance metrics of the ResNet-18-based Siamese network evaluated on measured calls. Training with early stopping at 1700 iterations yielded a cosine similarity of 0.9999 with a standard deviation of 8.8 × 10^−5^. Validation was conducted over 122 iterations using all pairs from the 30% validation subset, resulting in a cosine similarity of 0.9999 with a standard deviation of 9.6 × 10^−5^. These results confirm the model’s ability to reliably distinguish similarity and dissimilarity between chain signals, demonstrating both the robust feature extraction capability of ResNet-18 and the effectiveness of the positive/negative pair training strategy.

Table 4 presents the cosine similarity results evaluated using the trained ResNet-18-based Siamese network, comparing measured calls with reconstructed signals as well as with signals degraded to SNR levels of 15 dB and 10 dB. Pairwise similarity between individual chains was also assessed. The cosine similarity between measured calls and reconstructed signals averaged 0.9996, confirming a high degree of similarity within the latent feature space. These results indicate that the reconstructed signals not only reproduce the temporal and spectral characteristics of the measured calls but also preserve semantic similarity within the learned latent space. Noise robustness was further evaluated under low SNR conditions. With the same measured call as the reference, the mean similarity decreased progressively from 0.9720 at 15 dB to 0.8061 at 10 dB, demonstrating that signal similarity degrades with increasing noise.

In particular, at 10 dB SNR, chains 5, 7, 9, and 11 exhibited notably low similarity, indicating that even for the same signal, similarity can appear low under severe noise. This highlights the importance of reconstructing marine biological sounds under diverse environmental conditions (e.g., varying SNR levels) and building robust databases, as the lack of empirical recordings substantially hinders the development of data-driven approaches for acoustic signal analysis, species classification, bioacoustic modeling, and AI-based underwater acoustic technologies.

## 5. Summary and Discussion

This study presented a wavelet-based signal modeling framework to reconstruct the vocalizations of the brown croaker (*Miichthys miiuy*), integrating fk14 wavelet synthesis, parameter optimization using PSO, and deep learning-based validation. A key contribution of this study was the parameter-wise sensitivity analysis employing a normalized Bartlett processor, which identified the delay and scale (length) parameters as primary contributors to waveform similarity. This insight facilitated the establishment of biologically meaningful parameter ranges, and the synthesized waveforms closely reproduced the temporal and spectral features of the measured bioacoustic signals, including single-pulse morphology and IPI distributions. Additional statistical analyses, including 95% confidence intervals and residual error metrics, demonstrated that SPL values were faithfully preserved and IPI differences remained minor and biologically plausible.

Quantitative validation using a ResNet-18 based Siamese network yielded near-unity cosine similarity (average ≈ 0.9996) between measured and reconstructed signals, confirming the consistency of their latent representations within the learned feature space. Furthermore, similarity was evaluated under noisy conditions, with results showing a progressive decrease in cosine similarity as the SNR was reduced, indicating that the proposed reconstruction framework is affected by environmental noise. These results collectively confirm the proposed framework’s effectiveness in generating acoustically realistic and morphologically consistent fish vocalizations under data-limited scenarios.

Beyond signal synthesis, the proposed methodology holds substantial potential for applications such as bioacoustic dataset augmentation, improved training of AI-based classification systems, PAM, and species-specific call simulation. In future work, we will expand the proposed framework via the following:

1. Leveraging reconstructed signals as training data for generative models (e.g., GANs, WaveNet), enabling large-scale synthesis of biologically realistic fish vocalizations.
2. Explicitly incorporating environmental noise characteristics into the modeling and optimization process, either through preprocessing (e.g., denoising/whitening) or noise-aware objective functions, to enhance robustness under in situ conditions.
3. Extending the framework to additional vocal fish species and exploring its scalability for diverse marine bioacoustic applications.
4. Adapting the implementation for real-time field deployment, including potential GPU acceleration or embedded system integration for passive acoustic monitoring.

By addressing both signal fidelity and environmental robustness, the proposed framework provides a strong foundation for advancing bioacoustic modeling in real-world marine monitoring systems.

## Figures and Tables

**Table 1:** Optimal wavelet parameters obtained for each component used in waveform reconstruction.

Parameter	Waveform 1	Waveform 2	Waveform 3	Waveform 4	Waveform 5
Amplitude (A)	0.59	0.52	0.56	0.22	0.44
Delay (τ) × Fs	67	12	58	2	−11
Length (σ)	397	635	438	480	702

**Table 2:** Tabulated parameter intervals where Bartlett power remains above 0.98, indicating robust synthesis similarity for each waveform.

Parameter	Waveform 1	Waveform 2	Waveform 3	Waveform 4	Waveform 5
Amplitude (A)	0.14~0.99	0.3~0.76	0.33~0.95	0.0~0.83	0.26~0.59
Delay (τ) × Fs	60.8~69.8	9.6~16.6	50.8~65.8	−7.5~11.6	−15.6~−6.5
Length (σ)	363~448	612~652	408~473	388~547	677~746

**Table 3:** Cosine similarity and performance metrics of the ResNet-18-based Siamese network evaluated on measured calls.

	Similarity	Standard Deviation	Iteration
Train	0.9999	8.8 × 10^−5^	1700
Validation	0.9999	9.6 × 10^−5^	122

**Table 4:** Chain-wise cosine similarity between measured calls and reconstructed/SNR signals (based on ResNet-18 embeddings): similarities for reconstructed signals and for signals at SNRs of 15 dB and 10 dB, and the average.

Chain	Measured Calls—Reconstructed Signals	Measured Calls—SNR 15 dB	Measured Calls—SNR 10 dB
Average	0.999629	0.972008	0.806101
1	0.99998	0.997452	0.955398
3	0.999967	0.99634	0.934688
5	0.999968	0.981246	0.781015
7	0.999949	0.982604	0.773334
9	0.999952	0.932815	0.508469
11	0.999912	0.918859	0.704276
13	0.997679	0.994743	0.985527

## Data Availability

The data presented in this study are available upon request from the corresponding author. These data are not publicly available because they are part of an ongoing study.
